# Annotations

**Published:** 1892-04-09

**Authors:** 


### Annotations.

Readers of *Temple Bar* have found a little amusement in some of the incidents of the life of Benjamin Robert Haydon, a sketch of which is now appearing in that magazine. Haydon thought it sound policy to be on pleasant terms with his tradespeople, for which opinion he no doubt had reasons of his own. On one occasion he invited his butcher to make an inspection of the contents of his studio. This self-same butcher evidently had a soul above tripe. "I have a fancy for *genus*, sir," he said to the artist. "Mrs. Siddons, sir, ate my meat. Never was such a woman for chops. When she used to act that there character, you see—that there woman, sir, that murders a king between 'em—" "Lady Macbeth," suggested Haydon. "That's it, sir. . . . . I used to get up behind her carriage with the butler when she acted, and as I used to see her looking quite wild-like, and all the people frightened, 'Aha! my lady,' says I, 'if it wasn't for my meat, though, you wouldn't be able to do *that!*' You see, sir, I have fed Mrs. Siddons, John Kemble, Charles Kemble, Stephen Kemble, and Madame Catalini, sir, Morland, the painter, and —I beg your pardon—and *you*, sir. Madame Catalini, sir, was a wonderful woman for sweetbreads. But the Kemble family, sir, the gentlemen, rump steaks and kidneys in general was their taste. But Mrs. Siddons, sir, *she* liked chops!" This capital story has gone the round of the papers on its merits alone, and it seems a pity to spoil it with a moral. On the other hand, the every-day physiologist feels that it would be a still greater pity that so obvious and excellent a moral as it contains should be missed in these namby-pamby and "Bashkirtseffy" times. The moral is, that all those persons of genius of both sexes which this good butcher supplied with chops and sweetbreads, rumpsteaks and kidneys, had fine robust appetites; and we are quite inclined to agree with the butcher that the abundance of excellent meat with which he supplied them had really a very great deal to do with their splendid success in life. In this world the vigorous animal is almost an essential condition of the highest success of the mental and the spiritual. If our daily and weekly newspapers, and our monthly magazines and novels are becoming feebler, more captious, and less manly—and who shall say that they are not?—the probability is that one of the chief causes is the want of a good appetite and competent digestion for steaks and chops, sweetbreads and kidneys in their contributors. The thing is inevitable, gentlemen of the Press, the theatre, and the studio! If you cannot eat and enjoy yourselves like your professional ancestors, you will never be able to produce work so good as theirs.

**The Moral of Mrs. Siddon's "Chops."**

"Extraordinary treatment of a dying man." Such was the heading which the *Daily Chronicle*, one of the least sensational of our morning newspapers, placed over the report of the inquest which was held on the body of Patrick Dunn, who died of apoplexy at St. Bartholomew's Hospital last week. Probably most of our readers are already familiar with the facts of the case, but we will briefly recapitulate them. Dunn was fifty years of age, and of sober habits; he had been suffering from pains in the head, and had attended at St. Bartholomew's as an out-patient; he was subject to fits. A little before midnight on Friday, a city policeman in Cheapside heard a fall, and on turning round saw Dunn lying on his back in the road; he was insensible and bleeding from a wound at the back of his head. The policeman took Dunn to the hospital in a cab, and there the House Surgeon dressed his wounded head and ordered him back to the police-station, saying that he was drunk. Dunn had recovered a little consciousness by this time, and with the policeman's help managed to walk to the corner of the hospital, and then once more fell to the ground insensible. He was, however, taken to the police-station on an ambulance. But the inspector on duty, feeling anxious about him ordered him back to the hospital. Thither a second time he was taken; the House Surgeon again saw him, and used the stomach pump, but the man did not recover consciousness. Once more, in spite of his unconscious condition, he was ordered back to the police station, and then he was placed in a cell for the night. The inspector in charge, still dissatisfied, had him particularly well taken care of, and finally came to the conclusion that he was paralysed. At half-past six in the morning, Dunn, who had never recovered consciousness, was taken to the hospital by the police once more, for the third time, where he shortly died. It is plain from this recital that a physician of more experience than this youthful House-Surgeon might have acted very differently, and with a much juster appreciation of the real facts of the case. One's humanity is shocked by the idea of the five journeys this poor dying man was compelled to take between midnight and the break of day. Thrice was he taken to the apparently inhospitable hospital, and twice to the less cheerful, but more kindly and rational protection of the police cell. We do not wish to say anything to add poignancy to the unhappiness which the Bartholomew's House-Surgeon is now, without doubt, experiencing. But the system which makes such things possible will no longer satisfy the demands of a reasonable and increasingly kindly humanity. It is not right, and it is not necessary, that such things should be done. We cannot, of course, expect anything from a young man but youthful behaviour; and the young men of the present time, especially those who have risen to the elevation of House-Surgeons' appointments, must be excused if they are occasionally thrown off their mental balance by the extraordinary dignity to which they have successfully aspired. We must remember, too, as somebody has so justly observed, that newly-qualified medical men are always infallible. We repeat, therefore, that we do not blame the House-Surgeon so much as the system in vogue. A reform is imperatively demanded in the direction we have pointed out elsewhere in our present issue. The voluntary hospitals, of which, of course, Bartholomew's is not one, are now placed under the searching light of public criticism. But we are persuaded that it is for the most part kindly criticism, and, therefore, we are the more anxious that all the hospitals should bring up their methods to such a reasonable degree of efficiency as that their work may always be done without offending the sensibilities of an intelligent and not too exacting humanitarianism.

**The House Surgeon Question.**

A writer in the *Strand Magazine* has been narrating his impressions of the two methods of teaching the dumb a means of communication with their kind—audible speech and the language of the fingers. He seems to prefer the former, though he admits that in the institution he visited at Ealing he saw only its most favourable results. But the strength of a chain must be tried by its weakest link, and fascinating as the apparent miracle of the dumb speaking is to the popular mind, many who are engaged in instructing deaf mutes incline to think that the game is not worth the candle. How laborious the process of teaching deaf children to produce even the simplest sounds, the writer in the *Strand Magazine* shows very well; what he does not dwell on is the painful nature of the sound produced. It is articulate, but it is hardly human speech, but rather like the cry of some animal suffering and breathless. The voice tends even to go higher and higher up the scale, till a sentence begun at the average pitch ends with something like a shriek. Perhaps this may be a natural phenomenon enough, and we should all do the same—as instinctively we all walk in a circle when blindfolded—did not the ear correct the imperfections of the voice. But when the ear does not instruct the intonation, the result jars upon the listener. In many cases the necessity of acquiring speech is a real hindrance to a child's education. We have seen some who, using their fingers for communication, showed fair proficiency in history and geography, were good arithmeticians, and in every way seemed possessed of considerable intelligence; yet when it came to reading aloud, stumbled slowly and painfully over "A cat sat on a mat." Even those who show considerable ease in lip-reading, and write down passages accurately from dictation, seemed to suffer—there is no other word for it—when they had to speak. That the speech of a deaf mute could ever be mistaken for that of an ordinary person we doubt; and, therefore, doubt much its practical utility. It is an accomplishment which may now and then serve to make one or two seem—not feel, for lip-reading is all that is needed for this—more at ease in general company; but as a real help in the "struggle for life" it is in many cases utterly useless.

